# Endometrial microbiome during early pregnancy among women with and without chronic endometritis: a pilot study

**DOI:** 10.3389/fcimb.2025.1615182

**Published:** 2025-08-13

**Authors:** Hong Gao, Na Lu, Yahui Chen, Genlin Li, Huanhuan Li, Innie Chen, Amanda Black, Jenna Gale, Daniel J. Corsi, Xiaolan Wang, Kristin Connor, Shi Wu Wen

**Affiliations:** ^1^ Scientific Research Department, The Second Affiliated Hospital, Hengyang Medical School, University of South China, Hengyang, Hunan, China; ^2^ Ottawa Hospital Research Institute, the Ottawa Hospital, Ottawa, ON, Canada; ^3^ School of Nursing, University of South China, Hengyang, Hunan, China; ^4^ Center for a combination of Obstetrics and Gynecology & Reproductive Medicine, The First Affiliated Hospital, Hengyang Medical School, University of South China, Hengyang, Hunan, China; ^5^ Department of Gynecology, The Second Affiliated Hospital, Hengyang Medical School, University of South China, Hengyang, Hunan, China; ^6^ Department of Obstetrics and Gynecology, University of Ottawa, Ottawa, ON, Canada; ^7^ School of Epidemiology and Public Health, University of Ottawa, Ottawa, ON, Canada; ^8^ School of Epidemiology and Public Health, University of Ottawa Faculty of Medicine, Ottawa, ON, Canada; ^9^ Department of Health Sciences, Carleton University, Ottawa, ON, Canada

**Keywords:** endometrial microbiome, chronic endometritis, early pregnancy, 16S rRNA, host factors

## Abstract

**Introduction:**

Although chronic endometritis (CE) is strongly associated with infertility and adverse pregnancy outcomes, the specific microbiome of women with CE who can conceive remain unclear.

**Methods:**

This study recruited 100 participants aged 18 to 45 years with spontaneously conceived pregnancy who opted for pregnancy termination, detected their endometrial microbiome by 16S rRNA, and made a diagnosis of CE.

**Results:**

Among them, 19 were diagnosed with CE. There was a comparable microbial composition within the endometrium between women with and without CE. The relative abundance of *Sphingomonas* (21%) and *Pseudomonas* (8%) were the same in both groups. Compared to women without CE, women with CE exhibited higher abundance of *Faecalibacterium* (6.5% vs 3.8%), *Escherichia-Shigella* (3.3% vs 2.6%), Akkermansia (1.65% vs 1.1%), and lower abundance of *Lactobacillus* (10% vs 14%), and *Corynebacterium* (1.35% vs 2.15%) at the genus level. *Streptococcus*, *Escherichia-Shigella*, *Akkermansia* and *Finegoldia* exhibited significant interactions with other microbiome in participants with CE.

**Discussion:**

In women with CE, reproductive potential may be associated with the compositional stability of the endometrial microbiome, whereas an imbalance in the abundance of these microbes may be linked to their pregnancy outcomes.

## Introduction

Endometrium orchestrates a myriad of essential processes crucial to female reproductive health, encompassing menstruation, implantation, and successful pregnancy (Ang et al., 2023). Chronic endometritis (CE) is a persistent inflammation of the endometrium which can endure from preconception to pregnancy. (Morimune et al., 2022; Goto et al., 2023) CE is characterized by the presence of plasma cells within the endometrial/decidual tissue, and is associated with recurrent pregnancy loss (RPL), recurrent implantation failure (RIF), premature birth, abnormal uterine bleeding, and obstetric complications (Liu et al., 2018a; Cicinelli et al., 2019; Goto et al., 2023). In women of reproductive age, the incidence of CE was reported to be 10%~11%, while in the population with RPL and RIF, it can reach up to 60% (Cicinelli et al., 2014; McQueen et al., 2015; Puente et al., 2020). The risk of unexplained RPL caused by CE was astonishingly high, with odds ratio (OR) value of 24.90 (95% confidence interval (CI) 1.64-376.93) (Gay et al., 2021).

CE was often detected through an examination of endometrial or decidual tissue (Chen et al., 2023; Goto et al., 2023). Due to difficulties of obtaining endometrial samples, most studies to date have focused on female infertility population during fertility treatment, and it is largely unexplored what happens to the endometrial microbiome that has caused inflammation but remains asymptomatic after a woman conceives. We have therefore conducted a prospective cohort study comparing the endometrium microbiome of women with CE and those without in early pregnancy.

## Methods

### Study population

We conducted a cohort study at the gynecological clinic of the two large Affiliated Hospitals of Hengyang Medical School, University of South China in Hengyang, China between October 2021 and May 2022. Women aged 18 to 45 years who visited the gynecology clinics during the study period in early pregnancy and who conceived spontaneously with a normally developing fetus in their uterus but opt to terminate the unwanted or unplanned pregnancy were approached to participate in the study. The diagnosis of early pregnancy included amenorrhea or abnormal menstruation, positive serum or urine human chorionic gonadotropin (hCG), early intrauterine pregnancy (intrauterine gestational sac or germ appeared) confirmed by transvaginal color Doppler sonography (TVCD), and live fetus (primordial cardiac tube pulsation) (Xie et al., 2018). Based on the actual clinical diagnosis and treatment protocol for patients undergoing induced abortion in the gynecology departments of the two study hospitals, as well as expert consensus on perioperative female fertility protection during early pregnancy surgical abortion, the induced abortion operation was carried out at ≤9 weeks of pregnancy. At this stage of pregnancy, the operation not only had less surgical injury, less bleeding, and low risk of serious complications, but also offered a relatively high likelihood of successful removal of embryo-related tissue in one attempt (Costescu and Guilbert, 2018). Women with recorded use of antibiotics, hormones or immunosuppressive drugs within one month of conception, or with cervical treatment within one week or with vaginal flushing or local vaginal medication within 5 days or had sex within 48 hours before visiting clinic were excluded. Women with abnormal vaginal bleeding, gross vaginal inflammation, severe pelvic adhesion, uterine fibroids, endocrine disease, acute inflammation, cancer, sexually transmitted diseases, autoimmune disease, or severe heart, brain, liver or kidney diseases, or had incomplete clinical information were also excluded. According to the current research reports on the endometrial microbiota, when the sample size was over 40, the rarefaction curve indicated that a sufficient number of microbial taxa had been detected, and the taxa diversity in the samples had been fully captured. This study was performed in accordance to the Declaration of Helsinki and approved by the Ethics Committee of the University of South China [USC202108per01, August 24, 2021]. All participants fully understood the purpose of the study, were willing to participate in the study, and provided written informed consent. Moreover, this cohort study adhered to the STrengthening the Reporting of OBservational studies in Epidemiology (STROBE) reporting guidelines.

### Diagnosis of CE

Endometrium specimens were collected from all study participants, fixed in a 10% formalin solution, and subsequently embedded in paraffin. The immunohistochemistry staining for syndecan-1 (CD138) in the diagnosis of CE was conducted at the Pathological Diagnosis Laboratory of the University of South China. CD138-positive cells found in the endometrial stroma were defined as plasma cells. The diagnosis of CE was established when two senior pathologists independently identified five or more plasma cells in the endometrium under 10 high-power fields (HPF). Further details of the participants recruitment and grouping are provided in **Supplementary Figure 1**.

### Endometrial tissue collection and processing for laboratory investigation of microbiome

Endometrial tissue was collected during induced abortion. To ensure the representativeness of the sample and to prevent contamination, a specific procedure was implemented. First, the surgical procedure strictly adhered to the principle of maintaining sterility. Second, the cervix and vagina were fully exposed and thoroughly disinfected. Third, endometrial specimens were collected using a disposable sterile negative pressure suction probe guided by B-ultrasound prior to the removal of the embryo and endometrial tissue. The disposable sterile negative pressure suction probe entered the uterine cavity without negative pressure, aspirated endometrial tissue into the probe, promptly folded back the negative pressure tube, gently withdrew the probe from the genital tract, and took out the endometrial tissue specimen. The entire operational process avoided the probe from contacting the cervix and vaginal walls. Endometrial specimens were rinsed with normal saline and then divided into four disposable sterile cryopreservation tubes (NEST, 607001). One fresh tissue specimen was utilized for immunohistochemistry staining to detect CE, while the remaining specimens were stored in an ultra-low temperature (−80°C) refrigerator for next steps.

### 16S rRNA amplicon sequencing procedures and processing

The endometrial tissue was thawed and vortexed completely to resuspend the specimen in 2mL of PBS (pH=7.0). The suspension of each specimen was centrifuged at 12000 rpm for 5 minutes. The supernatant was removed, and the sediment was retained. The genomic DNA was extracted using the CTAB method (Nobleryder, CN), and subsequently evaluated for its purity and concentration by means of agarose gel electrophoresis. DNA was transferred to a centrifuge tube and then diluted to a concentration of 1ng/μL using sterile water for PCR amplification. PCR amplification using specific primers (341F and 806R) revealed the hypervariable V3-V4 regions of the 16S rRNA genes. PCR products were detected through agarose gel electrophoresis, and purified using qiagen gel extraction kit (Qiagen, Germany).

### Bioinformatic analysis

The Quantitative Insights into Microbial Ecology (QIIME) platform, available at https://qiime2.org/, was utilized for processing sequencing data (Bolyen et al., 2019). The FLASH software (V 1.2.11), found at http://ccb.jhu.edu/software/FLASH/, was employed for splicing the reads of the specimen in order to obtain raw tags (Magoc and Salzberg, 2011). The fastp software (V 0.20.0) was used for quality control of the raw tags in order to obtain high-quality clean tags. Vsearch (V 2.15.0) was used to compare clean tags with the Silva database (https://www.arb-silva.de/) and to identify the chimera sequences, thereby obtaining the effective tags (Moreno and Simon, 2018). Effective tags were denoised with DADA2 or deblur module in the QIIME2 software to obtain initial amplicon sequence variants (ASVs), and then filtering out ASVs with abundance less than 5 to get final ASVs and feature table (Koedooder et al., 2019). The QIIME2 software (specifically the classify-sklearn module) was used to compare final ASVs with the database to reveal the species information of each ASV. 16S V3-V4 region was annotated through the Silva138.1 database. Based on the ASV annotation results and the feature tables of each specimen, absolute and relative abundance tables of were generated at the kingdom, phylum, class, order, family, genus, and species levels.

### Statistical analysis

To investigate diversity of endometrial microbiome between CE and non-CE participants, alpha diversity metrics were calculated using R software (v4.2.1, vegan package, nonparametric two-tailed Wilcoxon rank-sum tests, *P*<0.05), including the Shannon diversity index and Chao index; beta diversity metrics were also conducted using R software (v4.2.1, vegan package, adonis, 999 permutations, *P*<0.05) based on Bray-Curtis distances, including principal coordinate analysis (PCoA) and permutational multivariate ANOVA (PERMANOVA) (Dixon, 2003; Anderson, 2008). Statistical significance was defined as a *P*-value less than 0.05.

The analysis of microbial data was conducted on a network platform called Wekemo Bioincloud (https://www.bioincloud.tech). The biomarkers at the genus level in both CE and non-CE participants were identified through linear discrimination analysis (LDA) as implemented using LEfSe (R, DESeq2 package, Kruskal Wallis *H*-tests, FDR<0.05, LDA score>4.0) (Brown et al., 2019). The interactions between endometrial microbiome and host factors were analyzed using spearman rank correlation (R, igraph package, Bray-Curtis, 478 complete linkage; SPSS 26.0) (Pang et al., 2020). The differences in the mean proportion of endometrial microbial taxa were compared using statistical analysis of metagenomic profiles software (STAMP, v2.1.3) (Parks et al., 2014). Significant differences in microbial taxa were observed using extended error bar plots, with statistical analysis conducted through Welch’s *t* test or Kruskal-Wallis *H* test (FDR<0.05). To account for the increased risk of false positives arising from multiple comparisons, all resulting *P*-values were adjusted using the Benjamini–Hochberg (BH) procedure for the False Discovery Rate (FDR) correction. An FDR-adjusted *P*-value less than 0.05 (FDR < 0.05) was considered statistically significant.

## Results

### Demographic and clinical characteristics of study participants

A total of 100 women aged 18–45 years with gestational ages ≤9 weeks were recruited into this study. Among them, 19 were diagnosed with CE. **Table 1** presents the distribution of demographic and clinical characteristics. The mean age of the participants was 31.10 ± 6.90 years, with 53% having completed junior college or higher education, and the median (IQR) gestational age was 6 (McQueen et al., 2015; Puente et al., 2020) weeks, and the median BMI was 19.47 (21.13-23.06) kg/m^2^. No statistically significant differences were observed in demographic and clinical factors between CE and no-CE participants.

**Table 1:** Demographic and clinical characteristics of the study participants, Hengyang, China, 2023.

Characteristics	Participants with CE (n=19)	Participants without CE (n=81)	Total	*P* value
Age, years	32.63 ± 5.91	30.74 ± 7.1	31.10 ± 6.90	0.285
Gestational Week (IQR)	6 (6-7)	6 (6-7)	6 (6-7)	0.732
BMI (IQR), kg/m^2^	20.96 (19.83-22.19)	21.36 (19.22-23.27)	19.47 (21.13-23.06)	0.857
Educational level				0.971
Senior high school or lower (≤9 years)	47.4% (9/19)	46.9% (38/81)	47% (47/100)	
Junior college or higher (>10 years)	52.6% (10/19)	53.1% (43/81)	53% (53/100)	
Household income (Chinese Yuan/month/person)				0.427
<5000	47.4% (9/19)	34.6% (28/81)	37% (37/100)	
5001	26.3% (5/19)	42.0% (34/81)	39% (39/100)	
≥8001	26.3% (5/19)	23.5% (19/81)	24% (24/100)	
Age of first sexual intercourse, years				0.077
≤20	36.8% (7/19)	59.3% (48/81)	55% (55/100)	
>20	63.2% (12/19)	40.7% (33/81)	45% (45/100)	
Frequency of sexual activity,/week				0.522
0	68.4% (13/19)	60.5% (49/81)	62% (62/100)	
≥3	31.6% (6/19)	39.5% (32/81)	38% (38/100)	
Number of sexual partners				0.932
1	63.2% (12/19)	64.2% (52/81)	64% (64/100)	
≥2	36.8% (7/19)	35.8% (29/81)	36% (36/100)	
Number of pregnancies				0.856
1	26.3% (5/19)	28.4% (23/81)	28% (28/100)	
≥3	73.7% (14/19)	71.6% (58/81)	72% (72/100)	
Number of deliveries				0.874
0	47.4% (9/19)	49.4% (40/81)	49% (49/100)	
≥2	52.6% (10/19)	50.6% (41/81)	51% (51/100)	
Number of induced abortions				0.758
0	42.1% (8/19)	38.3% (31/81)	39% (39/100)	
≥1	57.9% (11/19)	61.7% (50/81)	61% (61/100)	
History of pelvic surgery				0.264
Yes	68.4% (13/19)	54.3% (44/81)	57% (57/100)	
No	31.6% (6/19)	45.7% (37/81)	43% (43/100)	

### Composition of endometrial microbiome

The mean raw paired-end (PE) reads per sample was 82298 (range 62437 - 112133) in endometrial biopsy samples, with an average of 78013 (range 57019 - 99415) qualified reads and 73101 (range 45263 - 97742) nochime reads (**Supplementary Table 1**). Endometrium harbors a microbial community with low abundance. The detection rate of microbes in all samples of endometrial biopsy in this study was 100%.

**Figure 1:**
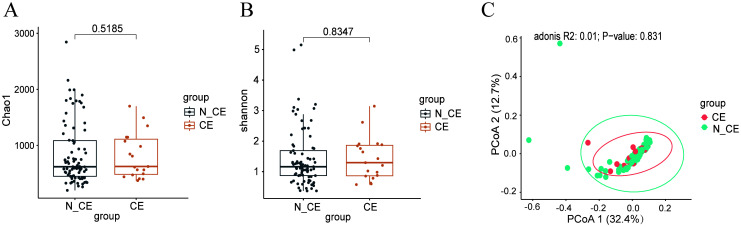
The diversity of the endometrial microbiome communities between participants with CE and those without (n=100) **(A, B)** the alpha diversity of endometrial microbiome **(A)**, Chao1 diversity index; **(B)**, Shannon diversity index) between participants with CE and those without. Box-plot elements consist of the median (represented by the center line), upper and lower quartiles (depicted as box limits), and 1.5×the interquartile range (shown as whiskers). **(C)** the beta diversity of endometrial microbiome was analyzed using Principal Coordinate Analysis (PCoA) between participants with CE and those without. PCoA was based on Bray-Curtis distances to analyze the microbial composition of endometrium. Each dot on the PCoA plot represents a single sample, with different colors indicating distinct sample groups. The 95% confidence intervals (CI) are represented by ellipses, *R^2^ =* 0.01, *P*=0.823. Statistical significance for correlations was determined using Spearman’s test with FDR correction (adjusted *P* < 0.05). N_CE, non-chronic endometritis; CE, chronic endometritis.

The microbial community diversity, including both alpha and beta diversity, was comparable between participants with CE and those without (**Figures 1A–C**). There was no statistically significant difference in the alpha and beta diversity of the endometrial microbiome communities between women with CE and those without (Chao1 index, *P*=0.79; Shannon index, *P*=0.66; PCoA, *R^2^ =* 0.01, *P*=0.82).

**Figure 2:**
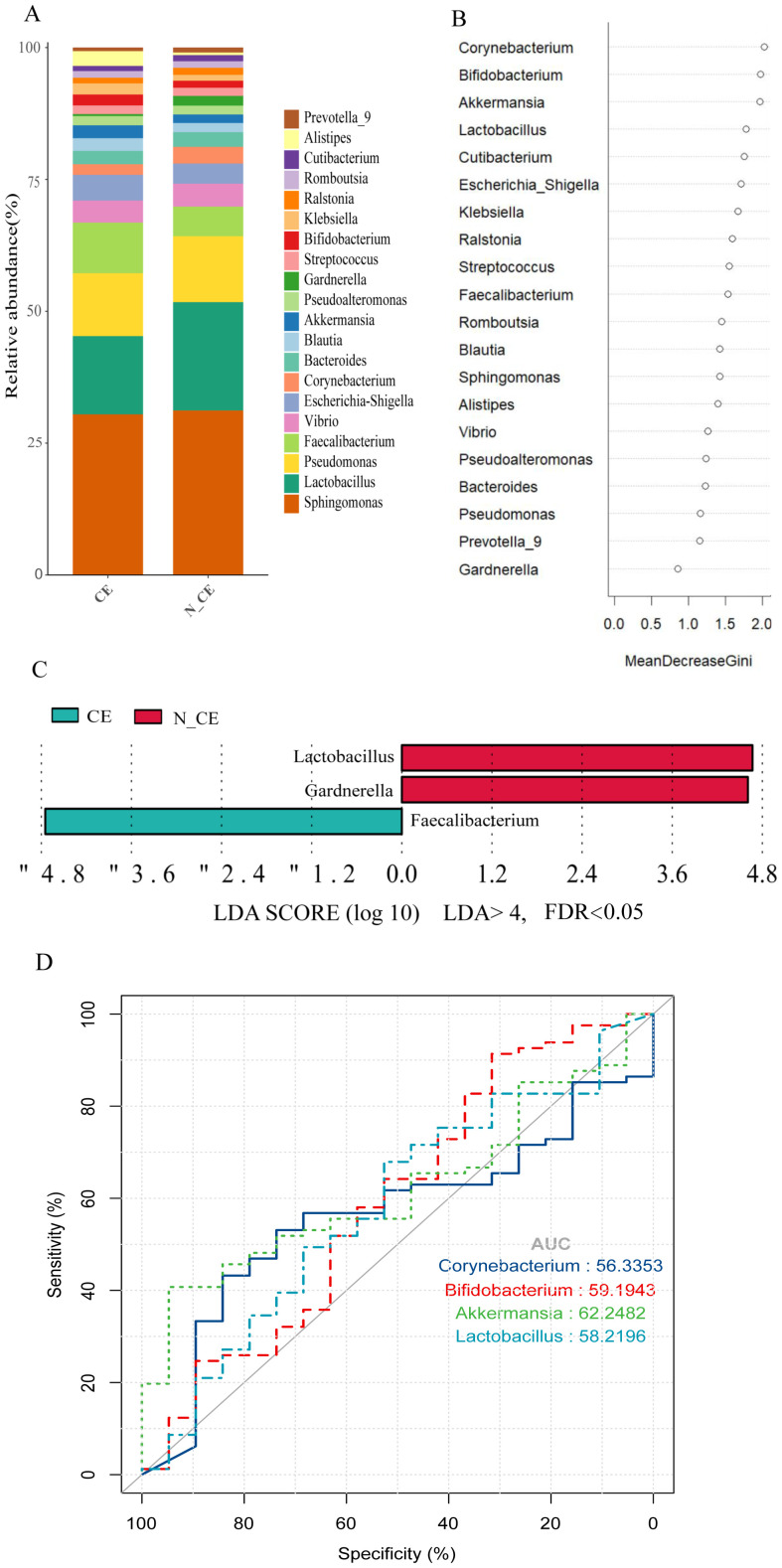
The composition, relative abundance, and differences of endometrial microbiome between participants with CE and those without **(A)** The composition of the top 20 microbial genera in the endometrium between participants with CE and those without. Each bar represents a distinct participant. **(B)** The rank of the Gini index from the random forest model. **(C)** Differences in the top 30 endometrial microbial genera between participants with and without CE. Linear discriminant analysis (LDA) and effect size (LEfSe) analysis were conducted to identify endometrial microbial biomarkers differentiating participants with and without CE. Features with an absolute log LDA score >4.0 and a false discovery rate (FDR)–adjusted *P*-value<0.05 (using the Benjamini–Hochberg correction) were considered statistically significant. CE, chronic endometritis; N_CE, non-chronic endometritis. **(D)** The ROC curves created by the potential biomarkers with relatively high Gini indices in B highlight the performance of the classifier at different cut-off points. The ROC curve visually describes the discriminative ability of the classifier by demonstrating the trade-off between sensitivity and specificity at different thresholds.

On the other hand, the relative abundance of the endometrial microbiome was different in participants with CE from those without (**Figure 2A**). *Lactobacillus, Sphingomonas*, *Pseudomonas*, *Faecalibacterium, Vibrio, Escherichia-Shigella, Corynebacterium, Akkermansia*, and *Klebsiella* were the major microbial genera. The relative abundance of *Sphingomonas* (21%) and *Pseudomonas* (8%) were the same in both groups. The relative abundance of *Faecalibacterium* (6.5%), *Escherichia-Shigella* (3.3%), and *Akkermansia* (1.65%) were higher and the relative abundance of *Lactobacillus* (10%) was lower in participants with CE. While in participants without CE, the relative abundance of *Faecalibacterium* (3.8%), *Escherichia-Shigella* (2.6%), and *Akkermansia* (1.1%) were lower and the relative abundance of *Lactobacillus* (14%), and *Corynebacterium* (2.15%) were higher (**Figure 2A**). Among them, *Lactobacillus* (*P*=0.036, FDR=0.039)*, Gardnerella* (*P*=0.021, FDR=0.039) *and Faecalibacterium* (*P*=0.039, FDR=0.039) were identified as the main biomarkers of endometrial microbiome (**Figure 2C**). Random forest model further identifed potential biomarkers, including *Corynebacterium, Bifidobacterium*, *Akkermansia* and *Lactobacillus*, with a relatively high Gini index for distinguishing between CE or N_CE (**Figure 2B**). The corresponding receiver operating characteristic(ROC) curves are shown in **Figure 2D**, which are potential markers of endometrial microorganisms in CE patients. The AUC values of *Corynebacterium*, *Bifidobacterium*, *Akkermansia* and *Lactobacillus* are approximately 0.56, 0.59, 0.62 and 0.58 respectively.

### Interactions of the endometrial microbiome in participants with and without CE

**Figure 3:**
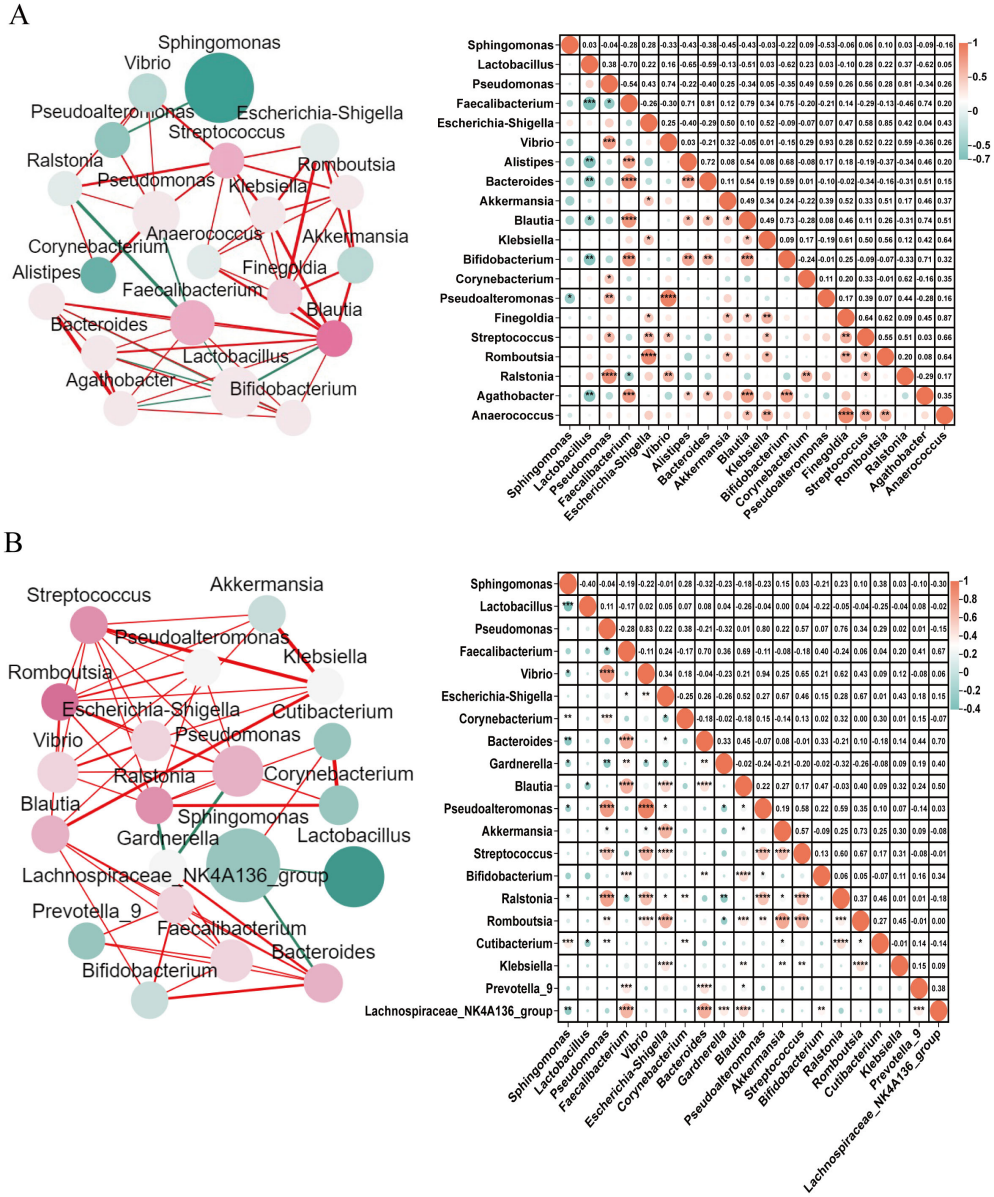
Co-occurrence networks of endometrial microbiome in participants with and without CE Co-occurrence networks of endometrial microbiome were drawn for the top 20 microbial genera in participants with **(A)** and without CE **(B)**. Each microbial genus network was established by calculating the co-occurring microbial communities with significant Spearman Correlation coefficients. In the network diagrams on the left, the circle size represents the standardized relative abundance; the colour of a node represents the degree of interactions between this node and other nodes, and the redder the colour, the more its interactions with other nodes; the thickness of the line between nodes represents the *P* value of Spearman Correlation, ranging from the most significant (thicker) to the least significant (thinner). Red lines indicate positive correlations and green lines indicate negative correlations. In the correlation graph on the right, the size of the circle and colour intensity are directly proportional to their corresponding Spearman correlation coefficients. No circle in a pair of microbiome means no correlation. * = significant correlations (Benjamini-Hochberg corrected *P*<0.05), *0.01<*P*<0.05, **0.001<*P*<0.01, ***P<0.001.

Endometrial microbiome co-occurrence networks were established to understand the interactions between different microbial genera in participants with and without CE (*r*>0.3, *P<*0.05, **Figure 3**). It can be seen that participants with CE showed a higher density and node degree distribution, which indicated more interactions between the endometrial microbiome. In participants with CE, *Sphingomonas* was the highest relative abundance, and S*treptococcus, Escherichia-Shigella, Akkermansia* and *Finegoldia* exhibited significant interactions with other microbiome (**Figure 3A**). In participants without CE, *Sphingomonas* also was the highest relative abundance, *Romboutsia* exhibited significant interactions with other microbiome (**Figure 3B**).

### Impact of host factors on the relative abundance of endometrial microbiome

**Figure 4:**
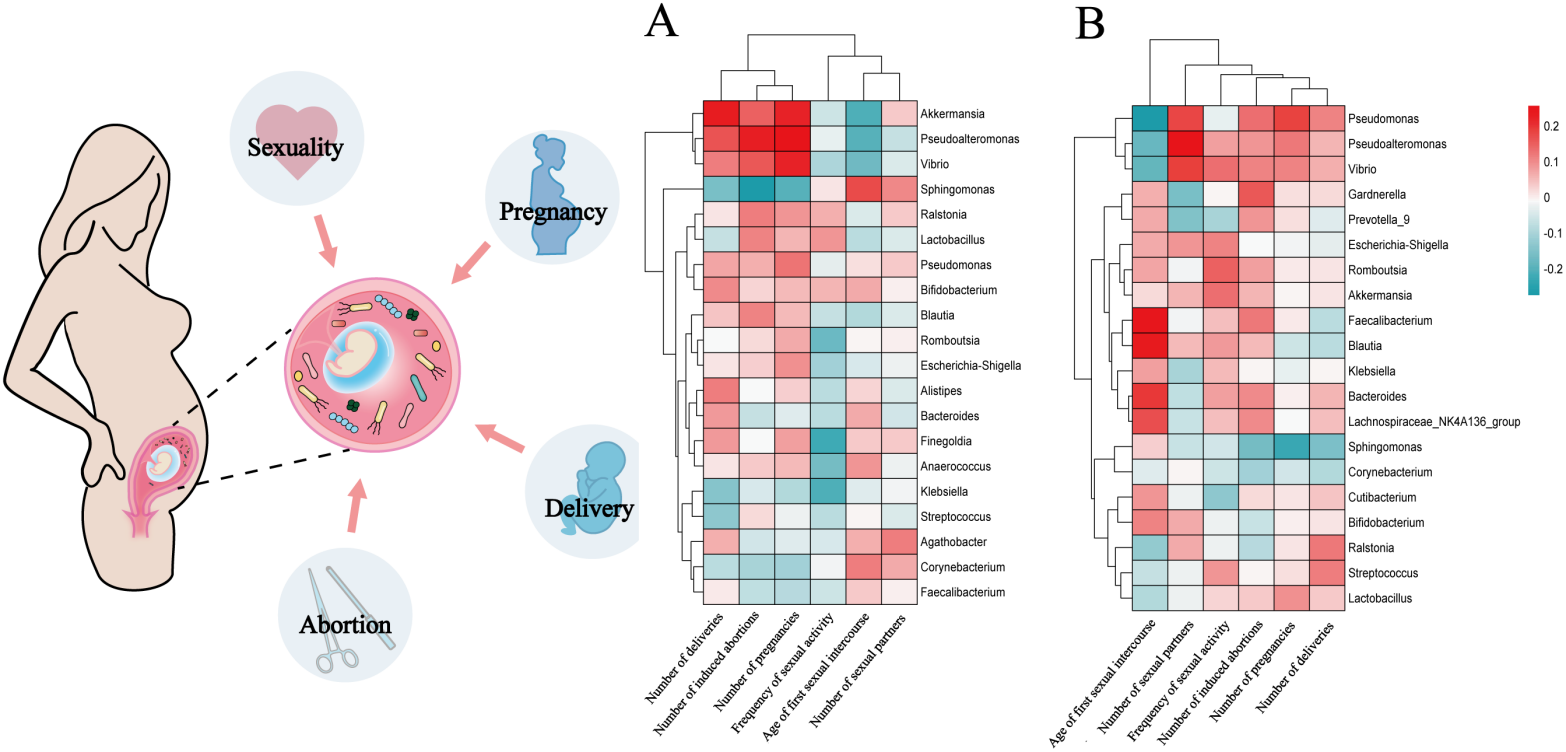
The relationship between the relative abundances of the top 20 endometrial microbial genera and host factors in participants with and without CE Each red square represents a positive correlation, while each green square represents a negative correlation. *P* values were acquired by the Spearman test. **(A)**, Correlations between the top 20 endometrial microbial genera and 6 host factors (120 comparisons) among participants with CE. **(B)**, Correlations between the top 20 endometrial microbial genera and 6 host factors (120 comparisons) among participants without CE. Multiple-hypothesis testing was corrected using the Benjamini-Hochberg method to control the false discovery rate (FDR).

The association between the relative abundance of the top 20 endometrial microbial genera and host factors are presented in **Figure 4**. Among participants with CE, the number of induced abortions exhibited a negative correlation with the relative abundance of *Sphingomonas* (*r*=-0.54, *P*=0.016, FDR=0.317); the number of induced abortions and pregnancies also demonstrated positive correlations with the relative abundance of *Pseudoalteromonas* (*r*=0.46 and 0.52; *P=*0.047 and 0.023; FDR=0.475 and 0.373, respectively); in addition, the number of deliveries was positively correlated with the relative abundance of *Akkermansia* (*r*=0.47, *P*=0.044, FDR=0.857) (**Figure 4A**). However, none of these associations remained statistically significant after FDR correction.

Among participants without CE, the age of first sexual intercourse positively correlated with *Faecalibacterium* and *Blautia* (*r*=0.25 and 0.24; *P*=0.026 and 0.032, FDR=0.213 for both) and negatively correlated with *Pseudomonas* (*r*=-0.27, *P*=0.013, FDR=0.213); the number of sexual partners had a positive correlation with the relative abundance of *Pseudoalteromonas* (*r*=0.26, *P*=0.021, FDR=0.420); the number of pregnancies exhibited a negative correlation with the relative abundance of *Sphingomonas* (*r*=-0.23, *P*=0.039, FDR=0.780) (**Figure 4B**). Similarly, these correlations did not reach statistical significance after FDR adjustment. Both the original and FDR-adjusted *P*-values are presented in **Supplementary Table 2**.

**Figure 5:**
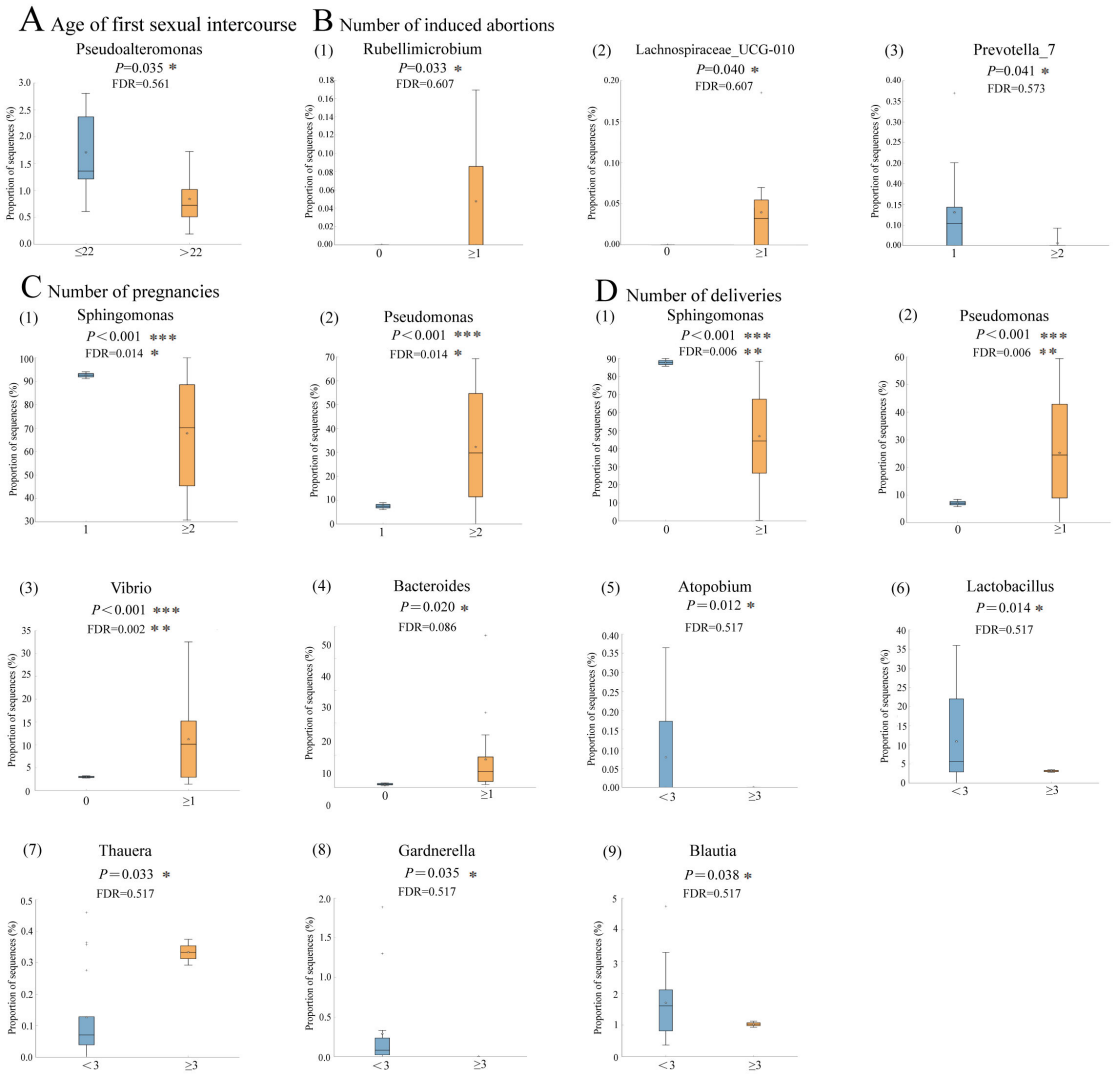
Comparative analysis on the relative abundance of endometrial microbiome under different host factors in participants with CE (n=19) **(A–D)**, the distribution and relative abundance of endometrial microbiome in patients with CE under different host factors. Box-plot elements include: median (center line), upper and lower quartiles (box limits), 1.5×interquartile range (whiskers); *P* values were determined by two-tailed Welch’s t test. Multiple-hypothesis testing was corrected using the Benjamini-Hochberg method to control the false discovery rate (FDR). *, *FDR*<0.05, **FDR<0.01, ***FDR<0.001.

Although not statistically significant after FDR correction, the prevalence of *Pseudoalteromonas* appeared to be higher in women who had their first sexual intercourse at or before age 22, suggesting a potential trend toward an association with early sexual debut (*P*=0.035, FDR=0.561, **Figure 5A**). Similarly, the relative abundance of *Prevotella_7* tended to be lower in women with ≥2 induced abortions compared to those with fewer abortions, although this difference did not remain significant after FDR adjustment (*P*=0.041, FDR=0.573, **Figure 5B**). When the number of pregnancies was ≥2, there was an observed increase in the relative abundance of *Pseudomonas* (*P*<0.001, FDR=0.014), while a decrease in the relative abundance of *Sphingomonas* was noticed (*P*<0.001, FDR=0.014, **Figure 5C**). When the number of deliveries was ≥1, the relative abundance of *Pseudomonas* (*P*<0.001, FDR=0.006)*, Vibrio* (*P*<0.001, FDR=0.002)*, and Bacteroides* (*P*=0.020, FDR=0.086) was significantly higher while a lower abundance of *Sphingomonas* was found (*P*<0.001, FDR=0.006). In contrast, when the number of deliveries reached ≥3, the relative abundance of *Lactobacillus* (*P*=0.014, FDR=0.517) *and Blautia* (*P*=0.038, FDR=0.517) showed a decreasing trend, although these changes were not statistically significant after FDR correction (**Figure 5D**). Comparative analysis on the relative abundance of endometrial microbiome across different host factors in participants without CE is shown in **Supplementary Figure 2**. A complete list of original and FDR-adjusted *P*-values is available in **Supplementary Table 3**.

## Discussion

For the first time, our study provided strong evidence from a prospective cohort design that endometrial microbiome composition was similar for women with CE versus those women who did not have CE in early gestation. On the other hand, major differences in relative abundance of endometrial microbes were observed between women with CE and those who without CE. The relative abundance of pregnancy-friendly microbes such as *Lactobacillus* decreased, while the relative abundance of opportunistic pathogenic microbes such as *Escherichia-Shigella* and *Akkermansia* increased in patients with CE as compared with those without.

Due to ethical concerns, it is impossible to collect tissue from endometrium of a healthy unpregnant women and follow them for conception and pregnancy outcomes. As a result, all human studies of the field, including the current one, have relied on assessment of tissue taken from endometrium in unhealthy or unwanted pregnancies. Despite a lack of tissue sample from human endometrium before the occurrence of pregnancy or diseases, the causation relation of bacterial infection and CE has been well established by animal models (Dohmen et al., 2000; Shokri et al., 2023) and by human studies showing effects of treatment of antibiotics for CE associated adverse outcomes including RPL, RIF, and infertility (Cicinelli et al., 2018; Cheng et al., 2022; Gu et al., 2023). What remains unknown is why some women affected by CE can conceive while others cannot, and why some women affected by CE can maintain a pregnancy and achieve live birth while others cannot. Our study, based on analysis of endometrium microbiome in women with spontaneously conceived pregnancy in early gestation, helps to shed lights on the potential mechanism here. The similarity in endometrial microbiome composition between women with and without CE suggested that the ability to conceive in women with CE may be related to a stable composition of their endometrial microbiota. In contrast, dysbiotic shifts in microbial abundance among women with CE may be linked to their pregnant outcomes. While longitudinal follow-up from conception to live birth was not conducted, previous studies suggested that progressive dysbiosis of the endometrial microbiota in women with CE might, upon reaching a critical threshold, lead to adverse outcomes such as early pregnancy loss (Fettweis et al., 2019; Moreno et al., 2022). Only one case report examined changes of dysbiotic in relative abundance of endometrial microbes from conception to end of pregnancy, and found that the *Lactobacillus* was decreased while *Gardnerella*, *Atopobium* and *Bifidobacterium* increased during the process, with the lowest *Lactobacillus* level observed when the pregnancy was lost (Garcia-Grau et al., 2019). CE is often found to be caused by bacterial infections and often leads to adverse reproductive outcomes, such as RPL, RIF, and unexplained infertility (McQueen et al., 2015; Cicinelli et al., 2018). Different types and proportions of endometrial microbiome can have varying impacts on female reproductive outcomes. We found that the relative abundance of *Faecalibacterium*, *Escherichia-Shigella*, and *Akkermansia* were higher and the relative abundance of *Lactobacillus* was lower in early pregnancy women with CE than in normal pregnancy. No previous study has reported the microbial communities in individuals with CE who conceived spontaneously and experienced no abnormal embryonic development in early pregnancy. Substantial evidence suggested that these microbes, such as *Alteromonas*, *Anaerococcus*, *Atopobium*, *Enterococcus, Escherichia, Klebsiella*, *Dialister, Bifidobacterium*, *Magasphaera, Parvimonas*, *Streptococcus, Prevotella, Propionibacterium*, etc., had been detected in CE patients with adverse reproductive outcomes (Chen et al., 2023; Moreno et al., 2018; Liu et al., 2019; Molina et al., 2020; Moreno et al., 2022). A lack of *Lactobacillus* and an increased *Streptococcus*, *Staphylococcus*, *Neisseria*, *and Klebsiella* were found in RIF women (Zou et al., 2023), and *Acinetobacter*, *Aliihoeflea*, *Anaerobacillus*, *Erysipelothrix*, *Bacillus*, *Hydrogenophilus* spp., *Staphylococcus*, and *Serratia* were prevalent in RPL women (Liu et al., 2018b; Liu et al., 2022). With regard to the changes in the abundance of endometrial microbes, the most commonly reported microbe was *Lactobacillus*. The term “dominant” was used to describe the absolute advantage of microbial abundance. Some studies suggested that the relative abundance of *Lactobacillus* could predict successful pregnancy outcomes, but there was still controversy over the setting of cut-off value, with some suggesting 90%, 80%, or 50%, or other values (Moreno I et al., 2016; Kyono et al., 2018; Moreno et al., 2022; Odendaal et al., 2024). When *Lactobacillus* were absent or near zero, it was often associated with poor reproductive outcomes (Moreno and Franasiak, 2017; Garcia-Grau et al., 2019; Zou et al., 2023). Drawing on our findings in early pregnancy and comparisons with previous studies on abnormal late pregnancies, it might be reasonable to speculate that a marginal imbalance in the abundance of endometrial microbiota could be associated with CE, yet still allow for spontaneous conception and early embryo development. However, more pronounced dysbiosis in microbial abundance, especially involving pathogenic bacterial invasion, was associated with adverse pregnancy outcomes such as CE, RPL, RIF, and others.

In CE patient’s endometrium, the interactions between certain potential opportunistic pathogenic microbes, such as *Akkermansia*, *Escherichia-Shigella*, and *Faecalibacterium*, became stronger, and they mutually promoted an increase in abundance. Accordingly, alterations in microbial abundance may be linked not only to the functions of specific microbes but also to their interactions with the abundance and functions of other microbes. The specific mechanisms underlying these interactions warrant further investigation. Based on our findings, we propose the following hypothesis that certain host factors, such as younger age at first sexual intercourse and a higher number of induced abortions or pregnancies, may be associated with changes in the relative abundance of endometrial microbes in participants with CE. No similar associations were observed in participants without CE. In participants with CE, some undesirable host factors were correlated with an increase in the relative abundance of non-optimal microbes. Most of the current research mainly focused on the influence of host factors on vaginal and cervical microbiome. Sexual behaviors, surgeries of the urinogenital tract, and pregnancy could modulate the vaginal and cervical microbiome communities (Francis et al., 2020; Artym and Zimecki, 2021; Lin et al., 2022). For instance, an early age of first sexual intercourse and a higher number of sexual partners were closely related to the colonization of non-optimal microbiome (Francis et al., 2020; Lin et al., 2022). However, no research on endometrial microbiome has been reported.

Notably, 57% of the participants in our study had a history of pelvic surgery, such as uterine evacuation or intrauterine device/balloon placement. This proportion is markedly higher than that reported in the general female population (typically 5–15%) and may limit the generalizability of our findings to all CE patients (Wang et al., 2022). However, this reflects the clinical reality of tertiary referral centers, where women with complicated reproductive histories are more likely to be evaluated and recruited. Surgical interventions have been shown to alter the reproductive tract microbiota through multiple mechanisms, including physical disruption of the cervical mucus barrier, promotion of microbial translocation from the vagina to the endometrium, and shifts in microbial-host interactions (Wang et al., 2022; Do et al., 2024). Previous studies have demonstrated that intrauterine devices and hysteroscopic procedures can significantly change microbial composition, favoring the colonization of non-optimal bacteria such as *Prevotella*, *Atopobium*, and *Streptococcus* in the upper reproductive tract (Wang et al., 2022; Do et al., 2024; Yagel et al., 2025). These changes may, in part, contribute to the dysbiotic microbial patterns we observed among CE patients in this study. Nevertheless, the inclusion of women with prior surgical history also offers valuable insights into a subgroup that may be particularly vulnerable to CE-related microbial dysbiosis and adverse outcomes. Our findings may thus provide clinically relevant information for reproductive-aged women with a history of surgical intervention who are attempting pregnancy.

## Strengths and limitations

There are several strengths of our study. It is for the first time that the endometrial microbial communities were examined in women with CE who conceived in a prospective design. Although CE was assessed in endometrial tissue after conception, but CE is likely a chronic process from pre-conception to early pregnancy, and what we observed in endometrial tissue after conception could reflect what happened before pregnancy. We examined endometrial tissue in early pregnancy without any known complications that may affect the endometrial microbiome and compared the microbiome of the endometrial tissue from women with CE to those of no CE women recruited from the same cohort with similar health status at the same stage of pregnancy. As a result, confounding by concomitant factors was reduced. However, limitations of our study should be acknowledged. First, we did not collect data on hormone levels in participants, so the potential impact of hormone on microbial communities could not be assessed. However, all participants were in the early stage of pregnancy (average gestational age of 6 weeks). At this stage of pregnancy, the variations in hormone levels may be limited (Whittaker et al., 2018). Second, the limited sample size is a recognized limitation of our study, which could potentially affect the generalizability and statistical power of the findings. The limited number of participants was primarily due to the strict inclusion and exclusion criteria, as well as the requirement for fresh and uncontaminated endometrial tissue samples for microbiome testing. While we strived to ensure that the selected participants were representative of the target population, the sample may not fully capture the heterogeneity of the broader patient population. Future studies with larger, more diverse cohorts are needed to validate these findings and enhance their applicability to clinical practice. Third, our study did not follow participants for long-term pregnancy outcomes, so the hypothesis that dysbiotic relative abundance of endometrial microbes in women affected by CE is related to adverse long-term pregnancy outcomes such as pregnancy loss cannot be tested directly in this cohort. Fourth, as samples were collected from two different tertiary hospitals, potential batch effects arising from differences in sample collection, transportation, and operating environments cannot be entirely ruled out. To ensure consistency, all participating researchers received standardized training in operations. Although multiple steps were taken to minimize variability, including following standardized protocols at both centers, transporting all samples to a central clinical research facility, processing and labeling them uniformly by the same researcher, storing them in a single –80°C freezer, and ensuring all laboratory procedures were performed by the same operator, some residual technical bias may still remain. To further minimize potential batch effects in future research, we plan to increase the sample size and enhance standardized training to ensure uniformity in sample collection procedures among all personnel. Fifth, a relatively high proportion (57%) of our participants had a history of pelvic surgery, which may introduce selection bias and limit the external validity of our findings. Surgical interventions are known to disrupt reproductive tract microbiota through changes in cervical barrier function and bacterial migration, potentially altering baseline microbial composition. As such, the microbial profiles identified in our cohort may reflect, at least in part, the impact of prior surgery. While this limits generalizability to all CE patients, it enhances the clinical relevance for a subset of women with complex reproductive histories. Finally, although 16S rRNA gene sequencing is a reliable method for microbial community testing and provides valuable taxonomic information, it also has limitations. The method is limited to bacterial profiling and excludes viruses and fungi. However, patients with confirmed fungal or viral infections, as detected through vaginal or cervical secretions, were excluded based on our inclusion and exclusion criteria. Furthermore, 16S rRNA sequencing lacks sufficient discriminatory power for precise species-level identification. Our analysis was centered on the genus level of microbial taxonomy.

## Conclusions

In early pregnancy, the overall composition of endometrial microbiome in women with CE was similar to that of healthy women; however, the relative abundance of specific microbes differed significantly. Adverse host factors may be associated with alterations in the relative abundance of endometrial microbes in women with CE, but not in those without CE. However, given the high proportion of participants with a history of pelvic surgery, these findings should be interpreted with caution, as they may not fully represent the general CE population.

## Data Availability

The original contributions presented in the study are included in the article/**Supplementary Material**, further inquiries can be directed to the corresponding authors.

## Supplementary material

Supplementary Figure 1 Flow chart of study participants and study process

Supplementary Figure 2 Comparative analysis on the relative abundance of endometrial microbiome under different host factors in participants without CE(n=81). A complete list of original and FDR-adjusted *P*-values is available in **Supplementary Table 4**.
