# Supplementary figures and images for: Endometrial microbiome during early pregnancy among women with and without chronic endometritis: a pilot study
Source: Front Cell Infect Microbiol. 2025 Aug 13;15:1615182. doi: 10.3389/fcimb.2025.1615182 (PMC12380780; doi:10.3389/fcimb.2025.1615182)

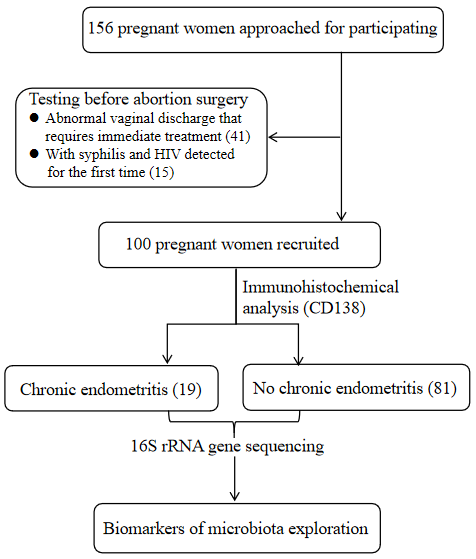

Supplement: Supplementary Figure 1 — Flow chart of study participants and study process [file Image1.tif]

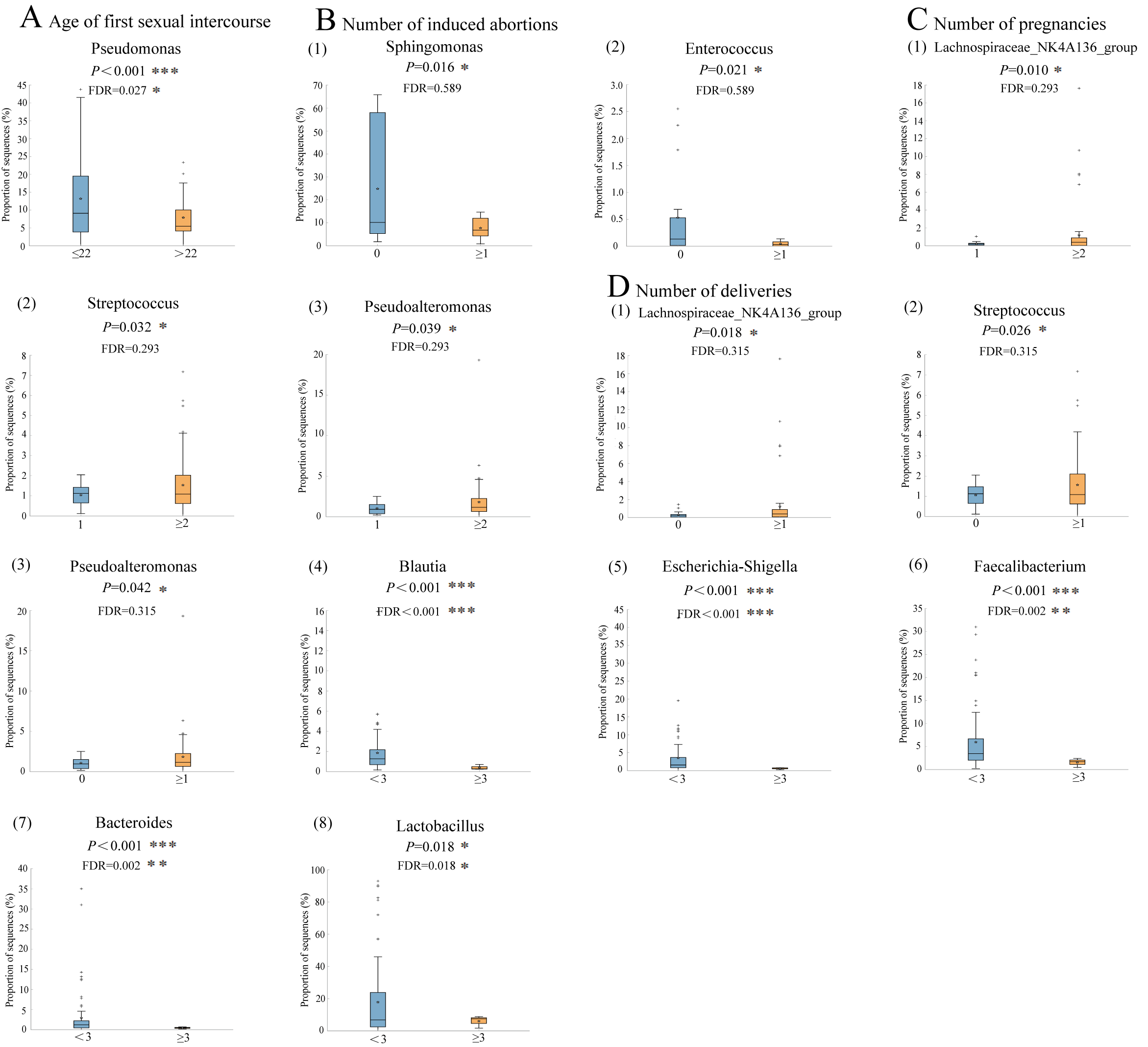

Supplement: Supplementary Figure 2 — Comparative analysis on the relative abundance of endometrial microbiome under different host factors in participants without CE(n=81). A complete list of original and FDR-adjusted P-values is available in Supplementary Table 4 . [file Image2.tif]
