# Supplementary material for: Endometrial microbiome during early pregnancy among women with and without chronic endometritis: a pilot study
Source: Front Cell Infect Microbiol. 2025 Aug 13;15:1615182. doi: 10.3389/fcimb.2025.1615182 (PMC12380780; doi:10.3389/fcimb.2025.1615182)
Supplement: Supplementary file 3 [file DataSheet1.pdf]

Supplementary Table 1. Decidual microbiota sequencing quality control

ample Nam	aw PE rea	mbined rea	lified re	Nochime reads	AvgLen(nt)	Q30	Effective%
CE1	74177	68261	67277	65531	389	96.24	88.34
CE2	87656	87393	86620	82212	388	96.37	93.79
CE3	81800	80345	79848	68384	394	96.44	83.6
CE4	74921	74801	74223	63368	396	96.3	84.58
CE5	85487	83118	82388	80776	381	96.48	94.49
CE6	80085	77719	76987	76148	383	96.33	95.08
CE7	88093	86832	86134	84752	374	96.57	96.21
CE8	85762	83348	82549	79394	372	96.34	92.57
CE9	89369	65494	64655	57186	379	96.29	63.99
CE10	81735	79799	78922	74888	395	96.24	91.62
CE11	85925	85668	84990	73696	390	96.4	85.77
CE12	83300	81128	80332	68804	392	96.33	82.6
CE13	88112	87756	86969	83183	386	96.47	94.41
CE14	85816	84963	84103	80134	386	96.48	93.38
CE15	75954	75928	75247	73494	386	96.39	96.76
CE16	77113	75718	74873	72398	385	96.3	93.89
CE17	81530	79820	78851	74916	389	96.11	91.89
CE18	112133	100125	99415	97742	376	93.86	87.17
CE19	88879	88656	87931	85901	388	96.36	96.65
N_CE1	77571	75342	74584	73281	385	96.36	94.47
N_CE2	79437	79048	78271	78080	381	93.56	98.29
N_CE3	87506	74111	73137	64104	377	96.25	73.26
N_CE4	86225	84168	83427	66007	393	96.45	76.55
N_CE5	89977	89587	88717	80363	389	96.32	89.32
N_CE6	73469	71092	70386	67894	383	96.55	92.41
N_CE7	72939	71443	70485	69040	397	96.08	94.65
N_CE8	86678	84790	83965	79010	384	96.44	91.15
N_CE9	82688	81662	80874	79588	387	96.1	96.25
N_CE10	77252	75615	74859	73681	383	96.52	95.38
N_CE11	78684	77252	76608	74747	383	96.43	95
N_CE12	84851	83084	82138	77721	401	96.21	91.6
N_CE13	75218	73724	72958	69259	388	96.23	92.08
N_CE14	80385	75403	74350	70878	392	96.19	88.17
N_CE15	73710	72774	72010	71725	381	93.77	97.31
N_CE16	84282	83850	83152	81645	384	96.41	96.87
N_CE17	80227	79289	78635	77473	382	96.59	96.57
N_CE18	79418	78835	78211	76823	383	96.39	96.73
N_CE19	83147	83073	82468	79805	386	96.24	95.98
N_CE20	86843	86012	85242	81089	386	96.56	93.37
N_CE21	84150	83124	82401	80905	375	96.45	96.14
N_CE22	75431	73069	72434	71313	379	96.35	94.54
N_CE23	76871	74535	73831	71995	382	96.34	93.66
N_CE24	80740	80185	79464	76349	377	96.43	94.56
N_CE25	83417	80392	79625	70627	384	96.64	84.67
N_CE26	81167	80596	79904	73843	391	96.61	90.98
N_CE27	87014	84787	83960	78038	386	96.44	89.68
N_CE28	85957	85785	85038	79355	386	96.39	92.32

N_CE29	76563	58362	57398	45263	374	95.52	59.12
N_CE30	88545	86445	85755	66442	392	96.48	75.04
N_CE31	77574	77245	76624	65212	387	96.2	84.06
N_CE32	77854	76504	75806	64829	394	96.26	83.27
N_CE33	79429	78942	78256	70651	387	96.47	88.95
N_CE34	74754	74721	74150	62614	394	96.51	83.76
N_CE35	76275	74787	74068	64457	392	96.31	84.51
N_CE36	82713	82028	81440	63099	397	96.24	76.29
N_CE37	83563	83531	82800	80583	386	96.42	96.43
N_CE38	85281	84424	83601	81928	385	96.34	96.07
N_CE39	78920	77031	76226	72774	398	96.19	92.21
N_CE40	82335	82291	81707	76681	390	96.53	93.13
N_CE41	100602	72558	71935	60968	370	93.85	60.6
N_CE42	76706	74749	73934	68721	395	96.37	89.59
N_CE43	76361	72541	71583	68638	386	96.36	89.89
N_CE44	81416	80908	80253	77234	381	96.4	94.86
N_CE45	74681	72722	72030	70393	379	96.4	94.26
N_CE46	76819	75739	74997	70943	387	96.32	92.35
N_CE47	74140	72958	72262	69951	387	96.25	94.35
N_CE48	78422	74681	73683	69689	387	96.31	88.86
N_CE49	62437	57796	57019	55025	384	96.47	88.13
N_CE50	83642	82707	81886	79363	387	96.42	94.88
N_CE51	77569	71267	70281	66941	377	96.4	86.3
N_CE52	88564	86571	85747	78092	390	96.52	88.18
N_CE53	80730	78928	78114	76242	386	96.23	94.44
N_CE54	74232	72557	71894	70092	385	96.46	94.42
N_CE55	84692	71153	70214	68660	382	96.32	81.07
N_CE56	84511	82468	81677	80002	376	96.39	94.66
N_CE57	84900	83105	82332	81876	379	96.53	96.44
N_CE58	88442	88350	87695	85843	377	96.48	97.06
N_CE59	83318	80983	80262	78469	380	96.47	94.18
N_CE60	74483	74094	73514	72244	377	96.34	96.99
N_CE61	81404	80637	80001	75421	387	96.47	92.65
N_CE62	81861	80632	80008	78574	362	96.54	95.98
N_CE63	80195	78191	77474	76666	381	96.32	95.6
N_CE64	78919	76343	75688	67941	383	96.63	86.09
N_CE65	88470	80500	79645	74269	385	96.53	83.95
N_CE66	83745	81466	80661	73599	386	96.45	87.88
N_CE67	71530	60063	59197	54808	384	96.48	76.62
N_CE68	81183	74552	73681	66225	387	96.3	81.57
N_CE69	80203	78977	78255	70944	384	96.48	88.46
N_CE70	77904	76208	75511	65352	392	96.51	83.89
N_CE71	74152	73252	72503	62543	391	96.25	84.34
N_CE72	88857	88163	87268	80209	394	96.15	90.27
N_CE73	86171	84826	83894	76272	388	96.37	88.51
N_CE74	85623	85014	84180	80826	386	96.53	94.4
N_CE75	78785	77135	76283	74212	387	95.91	94.2
N_CE76	81576	80185	79506	67663	391	96.3	82.94
N_CE77	101030	66681	66134	64896	359	93.58	64.23
N_CE78	109811	82066	81452	80113	371	94.14	72.96
N_CE79	99612	75434	74559	72309	360	88.22	72.59

N_CE80	84302	83274	82564	68368	391	96.44	81.1
N_CE81	80980	80972	80301	77450	389	96.4	95.64
Average	82298	78772	78013	73101	385	96	89
Total	8229887	7877216	7801356	7310124	38480	9613.36	8906.97
